# Subjective memory disorders and psychological distress in post COVID 19

**DOI:** 10.1192/j.eurpsy.2023.1659

**Published:** 2023-07-19

**Authors:** N. Halouani, D. Gdoura, O. Bouattour, A. Chamseddine, N. Moussa, S. Ellouze, J. Aloulou

**Affiliations:** 1 Psychiatry “B” Department; 2 Pulmonology Department, CHU Hedi Chaker, Sfax, Tunisia

## Abstract

**Introduction:**

In addition to psychological distress in patients with COVID 19, neurological and neurocognitive manifestations, such as memory impairment, are increasingly reported. Screening for cognitive impairment is therefore crucial.

**Objectives:**

Identify cognitive impairment inpost COVID19.

**Methods:**

This is a descriptive and analytical cross-sectional study that took place during the period from 1 st March 15 th May 2021 with 154 patients who were hospitalized at the COVID19 unit at Hedi Chaker Hospital Sfax. The psychometric evaluation, done by telephone, was performed using the &quot;Hospital Anxiety and Depression Scale&quot; for the screening of anxiety-depressive disorders, the &quot;Impact of Event Scale-Revised&quot; for the screening of post-traumatic stress disorder, the Insomnia Severity Index for the evaluation of sleep, the &quot;The Prospective and Retrospective Memory Questionnaire&quot; scale and the Mac Nair questionnaire for the evaluation of subjective memory.

**Results:**

The mean age was 66.62 ± 13.34 years. Male patients represented 60.4% of the population. The prevalence of anxiety, depression and post-traumatic stress disorder was 24.7%, 11% and 13.6% respectively. For the assessment of subjective memory, the mean total score of the PRMQ was 27.72 ± 7.71, with that of prospective and retrospective memory 15.41 ± 4.44 and 12.16 ± 3.73 respectively. According to the Mac Nair scale, 18.8% of patients had memory impairment (Mac Nair score &gt;15). Anxious patients showed more memory impairment. Depressed patients had the most impaired scores for total memory (p= 0.03) and retrospective memory (p= 0.022). Patients with post-traumatic stress disorder had more memory impairment (p=0.021).

**Conclusions:**

Psychological distress is multifactorial in its etiology. The medium and long term management of COVID+ patients must therefore be multidisciplinary.

**Disclosure of Interest:**

None Declared

